# Iodine concentration calculated by dual-energy computed tomography (DECT) as a functional parameter to evaluate thyroid metabolism in patients with hyperthyroidism

**DOI:** 10.1186/s12880-017-0216-6

**Published:** 2017-07-19

**Authors:** Duong Duc Binh, Takahito Nakajima, Hidenori Otake, Tetsuya Higuchi, Yoshito Tsushima

**Affiliations:** Department of Diagnostic Radiology and Nuclear Medicine, Gunma University Graduate School of Medicine, Faculty of Medicine, 3-39-22, Showa, Maebashi, Gunma 371-8511 Japan

**Keywords:** Dual energy, Computed tomography, Hyperthyroidism, Graves’ disease, Thyroid function, Scintigraphy, Iodine concentration

## Abstract

**Background:**

Thyroid function in patients with Grave’s disease is usually evaluated by thyroid scintigraphy with radioactive iodine. Recently, dual-energy computed tomography (DECT) with two different energy X-rays can calculate iodine concentrations and can be applied for iodine measurements in thyroid glands. This study aimed to assess the potential use of DECT for the functional assessment of the thyroid gland.

**Methods:**

Thirteen patients with Grave’s disease treated at our hospital from May to September 2015 were included in this retrospective study. Before treatments, all subjects had undergone both iodine scintigraphy [three and 24 h after oral administration of ^123^I (20 μCi)] and non-enhanced DECT. The region of interests (ROIs) were placed in both lobes of the thyroid glands, and CT values (HU: Hounsfield unit) and iodine concentrations (mg/mL) calculated from DECT images were measured. The correlation between CT values and iodine concentrations from DECT in the thyroid gland was evaluated and then the iodine concentrations were compared with radioactive iodine uptake ratios by thyroid scintigraphy.

**Results:**

Mean (±SD) ^123^I uptake increased from 46.3 (±22.2) % (range, 11.1–80.1) at 3 h, to 66.5 (±15.2) % (range, 40.0–86.1) at 24 h (*p* < 0.01). CT values ranged from 34.5 to 98.7 HU [mean: 67.8 (±18.6)], while the iodine concentrations calculated with DECT ranged from 0.0 to 1.3 mg/mL [mean: 0.5 (±0.4)]. A moderate positive correlation between CT values and the calculated iodine concentrations in the thyroid gland was seen (*R* = 0.429, *p* < 0.05). A significant negative correlation between ^123^I uptake at 3 h and iodine concentration by DECT were seen (*R* = −0.680, *p* < 0.05), although no correlation was observed between ^123^I uptake at 3 h and CT values (*p* = 0.087). No correlation was observed between ^123^I uptake at 24 h and CT values (*p* = 0.153) or that between ^123^I uptake at 24 h and iodine concentration by DECT (*p* = 0.073).

**Conclusion:**

The negative correlation of ^123^I uptake at 3 h with iodine concentration evaluated by DECT was better than that observed with simple CT value. DECT may have a potential role in the evaluation of iodine turnover in hyperthyroid patients.

## Background

Hyperthyroidism refers to a clinical state that results from excessive thyroid hormone levels. Typical symptoms include palpitations, tachycardia, fatigue, weight loss, and atrial fibrillation. The function of thyroid gland is regulated by thyroid stimulating hormone (TSH). Graves’ disease is the most common cause of hyperthyroidism, in which overstimulation of the thyroid gland by TSH receptor antibodies (TRAb) result in excessive production and release of thyroid hormones [1, 2].

Radioactive iodine (^131^I) is commonly used for treatment of hyperthyroidism [3–5]. Thyroid scintigraphy with ^123^I isotope is usually performed prior to ^131^I treatment to evaluate the functional status of the thyroid gland. For this purpose, patients are prescribed low-iodine diet for over 2 weeks, following which oral ^123^I is administered and its uptake at the thyroid gland assessed at 3 and 24 h (h). Patients with Graves’ disease typically show high iodine turnover and reduced iodine storage in the thyroid tissues, which results in decreased CT values of the thyroid glands [5]. Nygaard et al. reported a 50% decrease in ^131^I uptake 1 week after intravenous injection of 100 mL of iodine contrast agent, which suggests that iodine storage in the thyroid gland would be much affected by its uptake of radioactive iodine [6, 7]. These findings suggest that measurement of iodine concentration in the thyroid glands may be useful for the evaluation of iodine turnover, which is a key element of the metabolic pathway of thyroid hormone.

A previous study compared the use of CT and ^99 m^Tc-pertechnetate scintigraphy for evaluation of thyroid function in a feline model. The results showed that CT was a less sensitive modality than scintigraphy for functional characterization of thyroid gland [7]. Recent advances in CT technology allow for evaluation of iodine concentration with use of two images of the same slice acquired by dual energy CT (DECT) at different energy levels [8–12]. The use of two different energy levels allows the spectral decomposition of more than one element with a high atomic number [13, 14]. DECT incorporates various mechanisms to acquire two different energy images; these include dual-source, rapid-kV switching, and energy-sensitive detectors. The use of two different energy X-rays enables the estimation of effective atomic numbers, and to calculate concentrations of specific compounds [12–14]. Koonce, et al. reported an excellent correlation between iodine concentration measured by DECT and the true iodine concentration in a phantom study (mean measurement error < 3%) [15].

In the present study, we employed DECT with two different energy levels of 100 and 140 kVp for assessment of patients with Graves’ disease prior to ^131^I ablation therapy. We investigated the correlations between ^123^I uptake at the thyroid glands assessed with scintigraphy and the iodine concentration and CT values for thyroid glands acquired by DECT. The objective was to assess the potential use of DECT for functional assessment of the thyroid gland.

## Methods

### Patients

This retrospective study was approved by the institutional review board at the Gunma University Hospital. Thirteen consecutive female patients (mean age, 53.7 ± 13.2 years) underwent both iodine scintigraphy and DECT before radioactive iodine (^131^I) ablation therapy in the period between May 13th and September 8th, 2015. Inclusion criteria were: (a) Graves’ disease or hyperthyroid patients with a strong suspicion of Graves’ disease, and (b) iodine restricted diet for more than 2 weeks before iodine ablation therapy.

The diagnosis of Graves’ disease was based on clinical (signs of thyrotoxicosis such as tachycardia, weight loss, finger tremor, and excessive sweating; diffuse thyroid enlargement; and exophthalmos and/or specific ophthalmopathy) and laboratory [elevation in serum free thyroxine (FT_4_: mean, 1.70 ± 1.15 ng/mL; range, 0.67–5.38 ng/mL) and/or free triiodothyronine (FT_3_: mean, 5.90 ± 7.09 pg/mL; range, 2.28–30 pg/mL) levels, suppression of serum TSH (mean, 0.66 ± 1.29 μU/mL; range, 0.05–4.22 μU/mL); positive TRAb; or thyroid stimulating antibody (TSAb)] examinations.

### Image acquisition protocol

Thyroid scintigraphy was performed according to the institutional protocol. Oral ^123^I [dose: 20 μCi (7 MBq)] was administered. Scintigraphic planar images were obtained at 3 h and 24 h after administration with a conventional gamma camera system (E.CAM; Toshiba Medical Systems, Tokyo, Japan). A low-medium energy general purpose collimator was used (matrix size: 256 × 256; zoom, 2.0). The energy for ^123^I was set at 159 ± 7.5% keV. The scattered radiation estimate window was set to both sides of the photon peak window with a 7% window width. For scanning, thyroid phantom capsule with 3.7 MBq (0.1 mCi) was used; scanning time was 60 s for phantom, 10 s for background and 300 s for patient. For processing, square region of interest (ROI) was used. Background was also measured (longitudinal diameter: 1.2 cm).

Before radioactive iodine ablation therapy, basal thyroid gland volumetry was performed with dual energy technique with a dual source CT scanner (SOMATOM Definition flash; Siemens Healthcare, Forchheim, Germany). Scan parameters were: tube voltage, 140 kV and 100 kV; rotation time, 0.5 s; collimation, 14 × 1.2 mm; pitch, 0.9; effective tube current, 200/200 Quality ref. mAs; kernel, Q30 (SAFIRE strength 1); and slice thickness/interval, 3/1.5 mm.

### Measurement and data analysis

**Fig. 1:**
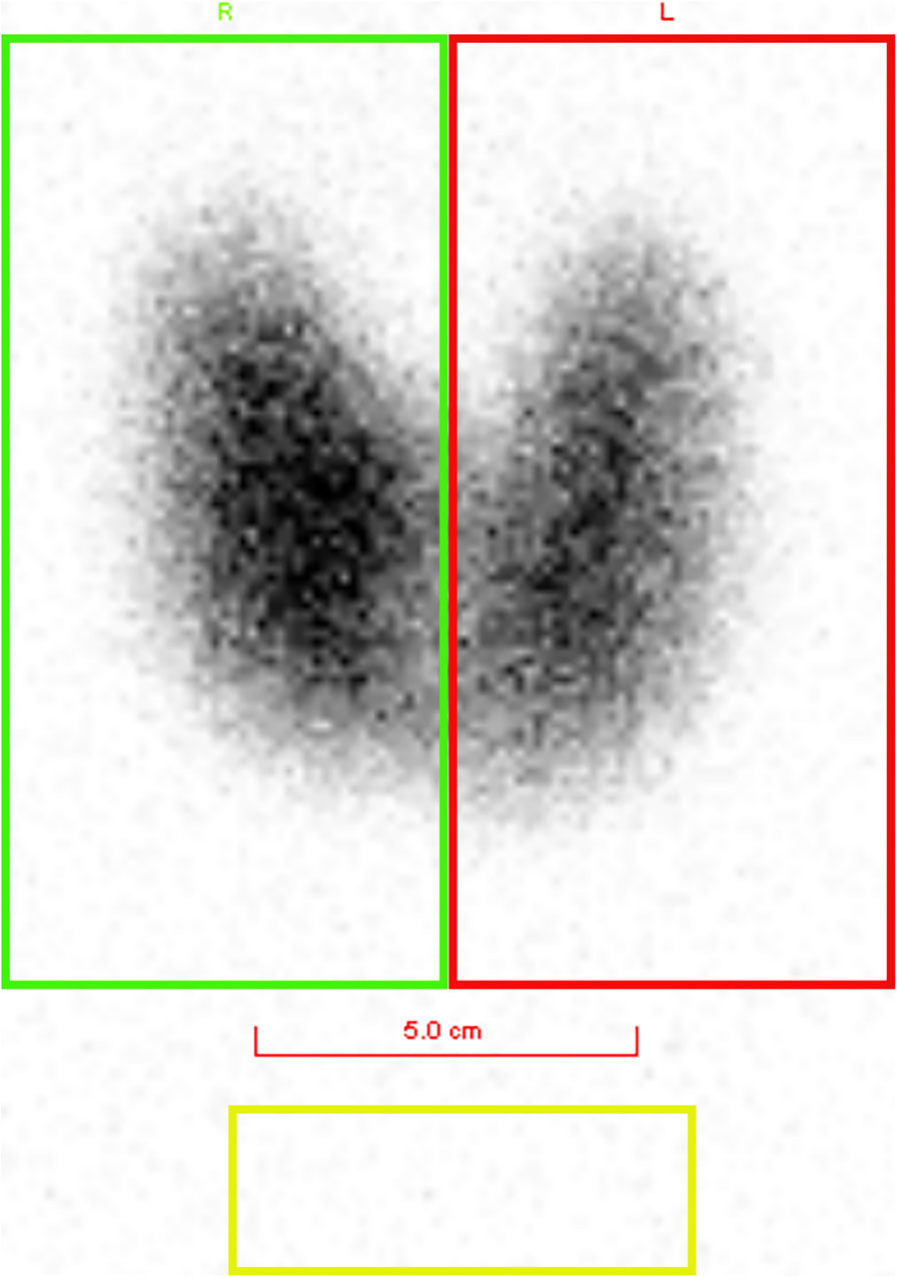
Thyroid scintigraphy showing measurements of ^123^I uptake: Square regions of interest (ROI) were used

For thyroid scintigraphy, ROIs were drawn around the borders of thyroid gland. ^123^I uptake was calculated as the sum of uptake at both lobes (Fig. 1). Total 13 thyroid uptakes were compared with DECT parameters.

For measurement of CT values and iodine concentration of the thyroid glands, CT data was transferred to a standard post-processing workstation (Syngo MMWP, VA 40 A, Siemens Healthcare, Forchheim, Germany). The data sets of CT images at different voltages for the same slices were analyzed to measure the iodine concentration with use of “brain hemorrhage” algorithm of the Syngo dual energy software. The iodine map image and the conventional 120 kVp images were generated from the low and high voltage CT data sets with slice thickness of 5 mm. The iodine concentrations and the CT values were measured from those images, respectively.

**Fig. 2:**
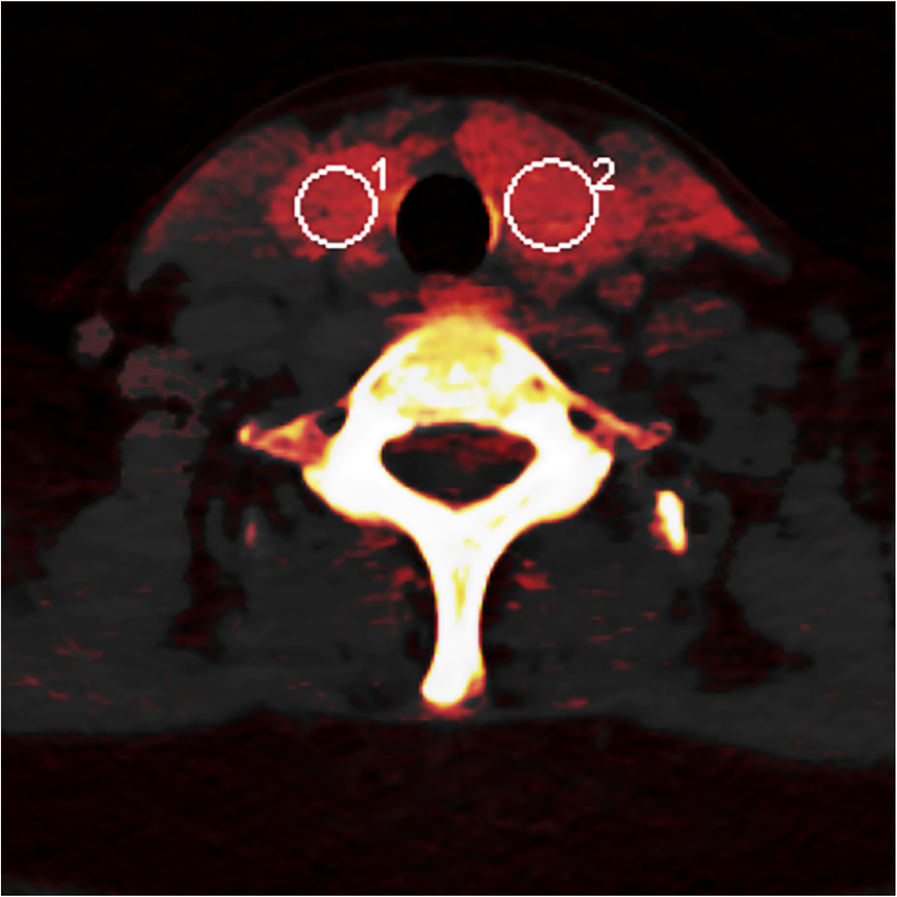
Fusion of two images of the same slice of the thyroid gland: iodine map and the composited 120 kVp image on DECT. ROIs are marked on both right and left lobes with minimal artifacts

The slices for the ROI setting were carefully selected with use of the following criteria: (a) minimal beam hardening artifacts; (b) homogenous area; and (c) no nodular lesions. We manually marked the ROIs on the right and left lobes of the thyroid gland. A largest possible ROI (round or oval-shaped) was marked taking care not to include the margins of the thyroid tissue. A total of 26 ROIs were included in the analysis (one each for the left and right lobes of the 13 patients; Fig. 2).

For assessment of the correlation between the CT value and the iodine concentration, each lobe was considered as a separate unit (*n* = 26). To assess the correlation of iodine concentration or CT value with ^123^I uptake at 3 h and 24 h, each patient was considered as a separate unit (*n* = 13).

### Statistical analysis

Statistical analysis was done using SPSS software (version 23; IBM-SPSS, Inc., IL, USA). The Spearman rank correlation coefficient was employed for linear correlation analyses. Wilcoxon signed-rank test was used to compare mean ^123^I uptake at 3 h and 24 h. *P* < 0.05 was considered statistically significant.

## Results

**Fig. 3:**
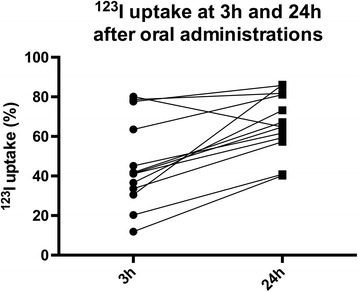
^123^I uptake at 3 h and 24 h after oral administration of 20 μCi: A significant increase in ^123^I uptake from 3 h to 24 h (*p* < 0.01)

^123^I uptake ratios in thyroid glands were increased in all but one patient. Mean ^123^I uptake increased from 46.3 ± 22.2% (range, 11.1–80.1) at 3 h to 66.5 ± 15.2% (range, 40.0–86.1) at 24 h (*p* < 0.01; Fig. 3). Mean increase in ^123^I uptake ratio from 3 h to 24 h was 143.6%.

**Fig. 4:**
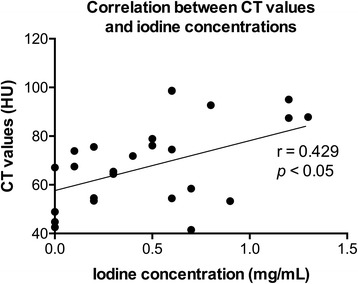
Correlation between CT values and iodine concentrations measured by DECT: A moderate positive correlation was noted (*R* = 0.429, *p* < 0.05). (HU: Hounsfield unit)

**Fig. 5:**
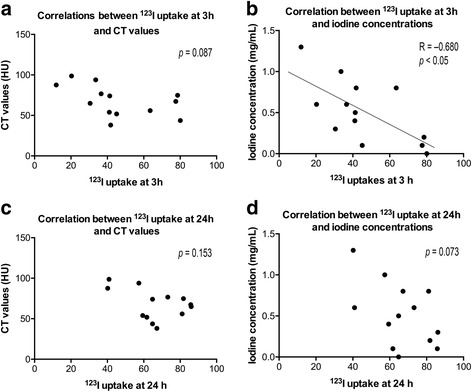
Correlation between **a** ^123^I uptake at 3 h and CT values; **b** ^123^I uptake at 3 h and iodine concentration on DECT; **c** ^123^I uptake at 24 h and CT values; and **d** ^123^I uptake at 24 h and iodine concentration on DECT

A moderate positive correlation between the CT values and the calculated iodine concentrations in the right and left lobes of the thyroid gland was seen (*R* = 0.429, *p* < 0.05, *n* = 26) (Fig. 4). The CT values ranged from 34.5 to 98.7 H.U. (mean ± SD: 67.8 ± 18.6), while the iodine concentrations calculated by DECT ranged from 0.0 to 1.3 mg/mL (0.5 ± 0.4). There was no correlation between ^123^I uptake at 3 h and CT values (*p* = 0.087; Fig. 5a), while a significant negative correlation between ^123^I uptakes at 3 h and iodine concentration assessed with DECT was observed (*R* = −0.680, *p* < 0.05; Fig. 5b). There was no correlation between ^123^I uptake at 24 h and CT values (*p* = 0.153; Fig. 5c) or that between ^123^I uptake at 24 h and iodine concentration (*p* = 0.073; Fig. 5d).

## Discussion

^123^I uptake at 3 h reflects the speed of uptake of iodine at the thyroid gland [16]. In our study, strong negative correlation was observed between ^123^I uptake at 3 h and iodine concentration on DCET. This result may reflect the higher capacity for both uptake and elimination of iodine by the thyroid glands, reflecting hyper metabolism of the thyroid glands. Particularly after iodine restricted diet, the elimination of iodine from the thyroid gland would be emphasized, resulting in low iodine concentration of thyroid glands by DECT. In other words, the iodine concentration may possibly reflect iodine turnover at the thyroid glands.

The correlation between ^123^I uptake and CT values of thyroid gland has not been investigated prior to the present study. Theoretically, a strong correlation between CT values and iodine concentration is plausible because iodine is the most important determinant of the CT values of the thyroid glands [17]. An experimental study that used polypropylene phantoms filled with different concentrations of iodine solutions [18] showed a strong correlation between CT value and actual iodine concentration. In our study, however, only a moderate linear correlation was observed between ^123^I uptake at 3 h and CT values of the thyroid gland in vivo, while the iodine concentration, as evaluated by DECT, showed a better correlation with ^123^I uptake. The reasons for these results are not entirely clear; however, there may be some possible explanations. First, hyperactive thyroid glands are usually hypervascular. These physiological changes are reflected in Doppler ultrasound studies. The increase in the vascular beds may have attenuated the CT values of the thyroid glands. Second, other pathological changes, such as edematous change may also contribute to the attenuation of CT values, although this aspect was not assessed in our study. We suspect that these factors may partially explain the stronger correlation between iodine uptake and iodine concentration assessed with DECT, rather than that with simple CT. Iodine concentration measurement by DECT may have a potential role in the evaluation of iodine turnover in hyperthyroid patients. Further investigation is necessary to elucidate the feasibility of this new technique.

The current DECT technique has an unavoidable drawback. The thyroid glands are located in the region of lower neck and upper mediastinum. The surrounding bones such as clavicle, sternum, and vertebrae may induce a beam hardening effect, which results in cupping and streaks artifacts [19]. It is highly likely that both CT value and iodine concentration measurement are affected by these artifacts. Ginat, et al. reported that DECT could reduce artifacts derived from metals and arteriosclerotic calcification in head and neck areas, and could potentially improve imaging quality [20]. In this study, the least affected CT slice was selected for measurement by visual inspection. However, the effect of inadequate image quality on our results cannot be ruled out.

There were some limitations of this study. Firstly, we had only 13 patients (26 ROIs). The correlation study showed much variability in each comparison. Secondly, we did not evaluate patient compliance to dietary restriction of iodine and relied on patients’ self-reported compliance. Furthermore, this study did not include other patients with euthyroid status, to evaluate the decrease in the ratio of iodine concentration on DECT scan. According to our hypothesis, the decrease in iodine concentration is liable to differ between patients with or without hyperthyroidism, when assessed with DECT before and after iodine restriction for 2 weeks. Thirdly, we did not compare the measured iodine concentration in the thyroid glands of our patients with true iodine concentration. The patients in our study were all candidates for radioactive iodine (^131^I) ablation therapy, thus no histopathological specimens were obtained. The iodine concentration may not be homogeneous, so the limited scope of the ROI may not have accurately reflected the iodine concentration.

## Conclusion

A significant negative linear correlation between ^123^I uptake at 3 h and iodine concentration calculated by DECT was observed. This result suggests that iodine concentration could predict thyroid function, similar to thyroid scintigraphy. Iodine concentration measurement by DECT may have a potential role in the evaluation of iodine turnover in the thyroid gland. Further studies with larger number of patients are required to confirm these results.

## Abbreviations

CT: Computed tomography
DECT: Dual energy computed tomography
FT3: Free triiodothyronine
FT4: Serum free thyroxine
HU: Hounsfield unit
ROI: Region of interest
TRAb: TSH receptor antibodies
TSAb: Thyroid stimulating antibody
TSH: Thyroid stimulating hormone
